# Development of near‐infrared firefly luciferin analogue reacted with wild‐type and mutant luciferases

**DOI:** 10.1002/chir.23236

**Published:** 2020-05-04

**Authors:** Nobuo Kitada, Ryohei Saito, Rika Obata, Satoshi Iwano, Kazuma Karube, Atsushi Miyawaki, Takashi Hirano, Shojiro A. Maki

**Affiliations:** ^1^ Department of Engineering Science, Graduate School of Informatics and Engineering, The University of Electro‐Communications Chofu Japan; ^2^ Center for Neuroscience and Biomedical Engineering The University of Electro‐Communications Chofu Japan; ^3^ School of Pharmacy Tokyo University of Pharmacy and Life Science Tokyo Japan; ^4^ Laboratory for Cell Function and Dynamics Center for Brain Science Saitama Japan

**Keywords:** Akaluc, luciferin analogues, luciferin‐luciferase reaction, mutant luciferase, near‐infrared bioluminescence, *Photinus pyralis* luciferase, TokeOni

## Abstract

Interestingly, only the *D*‐form of firefly luciferin produces light by luciferin–luciferase (L–L) reaction. Certain firefly luciferin analogues with modified structures maintain bioluminescence (BL) activity; however, all *L*‐form luciferin analogues show no BL activity. To this date, our group has developed luciferin analogues with moderate BL activity that produce light of various wavelengths. For in vivo bioluminescence imaging, one of the important factors for detection sensitivity is tissue permeability of the number of photons emitted by L–L reaction, and the wavelengths of light in the near‐infrared (NIR) range (700–900 nm) are most appropriate for the purpose. Some NIR luciferin analogues by us had performance for in vivo experiments to make it possible to detect photons from deep target tissues in mice with high sensitivity, whereas only a few of them can produce NIR light by the L–L reactions with wild‐type luciferase and/or mutant luciferase. Based on the structure–activity relationships, we designed and synthesized here a luciferin analogue with the 5‐allyl‐6‐dimethylamino‐2‐naphthylethenyl moiety. This analogue exhibited NIR BL emissions with wild‐type luciferase (*λ*_max_ = 705 nm) and mutant luciferase AlaLuc (*λ*_max_ = 655 nm).

## Graphical abstract

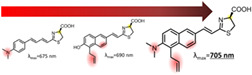

## 1 INTRODUCTION

**FIGURE 1:**
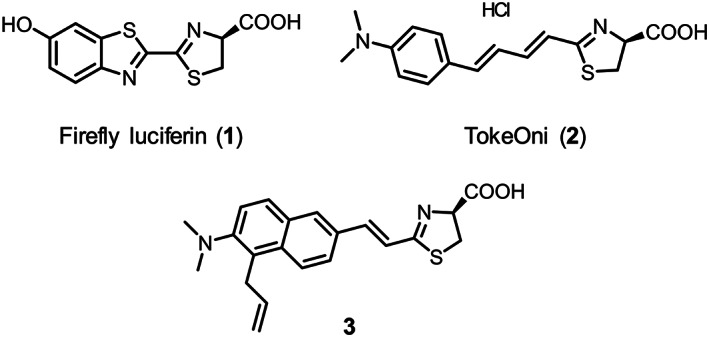
Structures of firefly luciferin (**1**), TokeOni (**2**), and designed luciferin analogue **3** [Correction added on 5 June 2020, after first online publication: The figure 1 image has been corrected.]

Firefly bioluminescence (BL) showed light emission caused by the reaction of firefly luciferin (**1**, Figure 1) catalyzed with firefly luciferase in the presence of Mg^2+^, in which **1** is first adenylated with ATP followed by the oxidative reaction with O_2_ to generate oxyluciferin with a yellow‐green light (*λ*_max_ = 560 nm).^1^, ^2^ This reaction is termed as the luciferin–luciferase (L–L) reaction. Firefly luciferin **1** and luciferase are biosynthesized in the body of firefly, and **1** has a chiral center at C3 with the same stereochemistry as unnatural *d*‐cysteine (*d*‐form). Interestingly, despite the fact that the *l*‐form of firefly luciferin has significantly low BL activity of L–L reaction^1^, ^3^, ^4^; however, we reported that the *l*‐form of firefly luciferin is able to produce light by conversion to *d*‐form **1** through the luciferyl‐CoA under the action of luciferase.^5^

The L–L reaction is applied to optical imaging techniques in the fundamental research fields of medical and biological sciences.^6^, ^7^, ^8^ One of the solutions to improve optical in vivo imaging technique is an increase in the permeability of light from deep site of biological tissue. Because the permeability of near‐infrared (NIR) light is higher than that of visible light (450–600 nm) for biological tissue,^9^, ^10^ researchers have been engaged in developing luciferin analogues^11^, ^12^ and mutant luciferases^13^ producing NIR light by the L–L reactions. These luciferin analogues and mutant luciferases successfully enabled high‐resolution optical in vivo imaging compared with the use of the wild‐type luciferin **1** and luciferase. Our group developed luciferin analogues producing light with various wavelengths,^14^, ^15^, ^16^, ^17^ and some of the analogues were tested for in vivo experiments. Then, we confirmed that the analogues enabled to detect light emission from the deep target tissue of mice with high sensitively.^18^, ^19^, ^20^ In addition, Aka‐BLI, which is the combination of a NIR luciferin analogue, TokeOni (**2**, Figure 1) with a mutant luciferase, Akaluc, produced NIR light and made it possible to detect the BL emission from the brain in a marmoset.^21^

**FIGURE 2:**
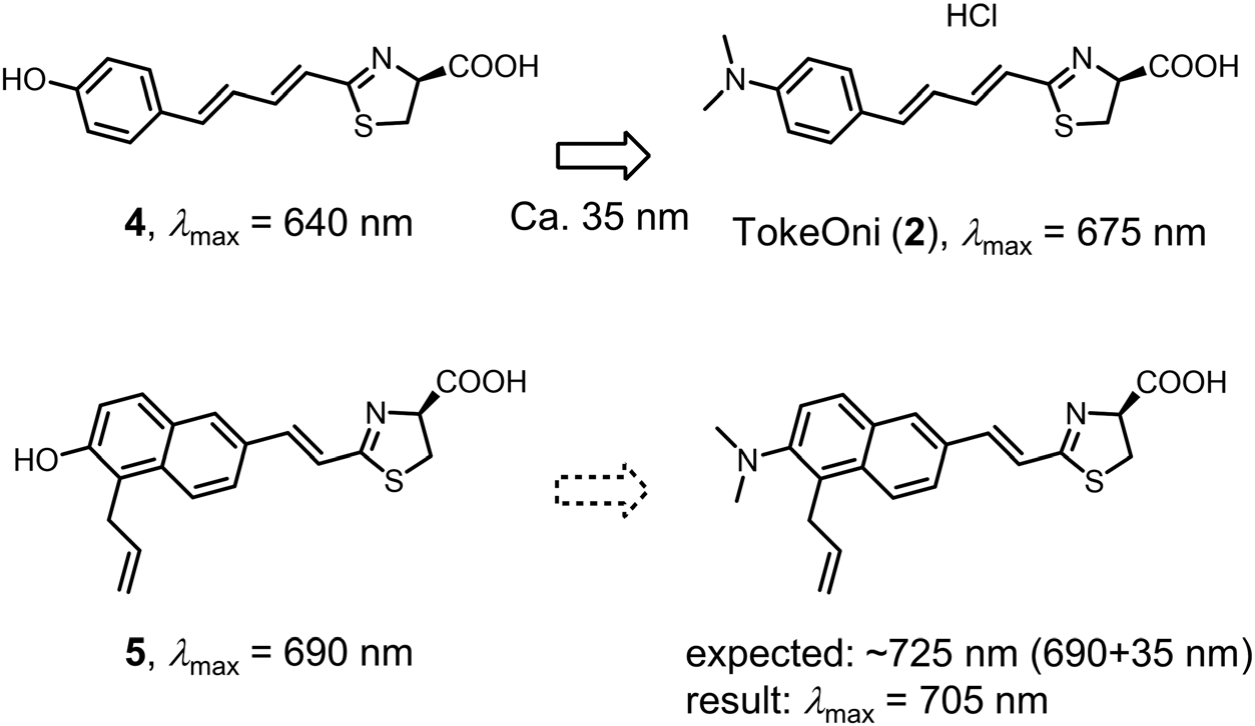
Structure–BL activity relationships for luciferin analogues. Analogues **2**, **4**, and **5** were previously reported^14^, ^17^

Although, there are a number of luciferin analogues, only limited analogues can produce NIR light (over 700 nm) reacted with wild‐type luciferase. To design a new luciferin analogue, we have evaluated a structure–BL activity relationship of our luciferin analogues for the wavelength of L–L reaction with wild‐type luciferase (Figure 2).^14^, ^17^ One conclusion of the evaluations lead us to design analogue **3** based on the data of **2**, **4**, and **5**. The BL emission maximum (*λ*_BL_) of **2** with the dimethylamino group is 35‐nm red shifted from that of **4** with the hydroxyl group, although **2** and **4** have the common phenyl‐1,3‐butadiene structure.^14^ When **4** and **5**, both of which contain the hydroxyl group, are compared, the *λ*_BL_ of **5** is 50‐nm red shifted from that of **4**.^17^ Hence, we designed **3** to have the 5‐allyl‐2‐naphthylethenyl moiety and a dimethylamino group at C6. The structure–BL activity relationship predicts that the *λ*_BL_ value of **3** will be 725 nm. In this report, we prepared **3** and investigated its BL activity with *Photinus pyralis (Ppy*) luciferase and Akaluc,^17^ comparing its properties to those of **1**, **2**, and **5**.

## 2 MATERIALS AND METHODS

### 2.1 General

Commercially available reagents and solvents were used without further purification. For bioluminescence measurements, TokeOni (**2**) was provided by Kurogane Kasei Co., Ltd. and recombinant *Ppy* luciferase (QuantiLum® recombinant luciferase, E1701, Promega) was used. Wako Silica gel 70 F254 thin‐layer chromatography plates were used for analytical thin‐layer chromatography, and Kanto Chemical Silica gel 60 N (spherical, neutral) was used for column chromatography. For preparative flash chromatography, an automated system (Smart Flash EPCLC AI‐580S, Yamazen Corp., Japan) was used with universal columns of silica gel. Melting points were measured with a Yanaco MP‐500P. IR spectra were obtained with a Nicolet 6700 spectrometer with an attenuated total reflection attachment.^1^H and ^13^C nuclear magnetic resonance (NMR) spectra were recorded on a JEOL ECA‐500 instrument (500 MHz for ^1^H and 126 MHz for ^13^C). High‐resolution electrospray ionization mass spectra were obtained with a JEOL JMS‐T100LC mass spectrometer. The optical purities of NIR analogue **3** was analyzed by high‐performance liquid chromatography (HPLC; Agilent 1100 series) using a Daicel chiral column (Daicel Chemical Industries, OD‐RH, 5 μm, 4.6 × 150 mm, flow rate 0.5 ml/min). Bioluminescence spectra were measured with an ATTO AB‐1850 spectrophotometer. Bioluminescence intensities were monitored using an ATTO AB‐2270 luminometer. Density functional theory (DFT) calculations were performed with the Gaussian 09 program (Rev. D.01).^22^ DFT included the B3LYP function with the 6–31 + G(d) basis set.^23^, ^24^, ^25^ Molecular graphics were prepared with GaussView, Version 5.^26^

### 2.2 Synthesis of NIR analogue **3**

#### 2.2.1 Bromoamine **3b**

A solution of 6‐amino‐2‐naphthoic acid methyl ester (**3a**) (5.35 g, 26.6 mmol) in dimethyl sulphoxide (DMSO; 50 ml) and *N*‐bromosuccinimide (4.84 g, 27.2 mmol) was added, and the mixture was stirred for 10 min at r.t. The reaction mixture was diluted with water and extracted with ethyl acetate (3 × 150 ml). The combined organic layers were dried over Na_2_SO_4_, filtered, and the solvent are removed under reduced pressure. The obtained residue was purified by silica gel column chromatography (hexane/ethyl acetate = 4/1) to yield bromoamine **3b** (6.97 g, 24.9 mmol, 93%) as a light brown solid: ^1^H NMR (500 MHz, CDCl_3_) *δ* 8.43 (d, *J* = 1.7, 1H), 8.06 (dd, *J* = 8.6, 1.7 Hz, 1H), 8.03 (d, *J* = 8.6 Hz, 1H), 7.70 (d, *J* = 8.6 Hz, 1H), 7.03 (d, *J* = 8.6 Hz, 1H), 3.96 (s, 3H); ^13^C NMR (126 MHz, CDCl_3_) *δ* 167.29, 144.31, 135.62, 131.30, 130.16, 127.46, 127.34, 125.09, 124.36, 118.24, 103.40, 77.38, 77.12, 76.86, 52.20; HR‐ESI‐MS: m/z: [M + H]^+^ calcd for C_12_H_11_BrNO_2_, 279.9973, 281.9953; found, 279.9931, 281.9910.401.

#### 2.2.2 Dimethylamine **3c**

To a solution of bromoamine **3b** (2.39 g, 8.54 mmol) in tetrahydrofuran (30 ml), sodium cyanoborohydride (2.63 g, 41.9 mmol) and formaldehyde (35% in H_2_O, 15 ml, 195 mmol) were added, and the mixture was stirred in an ice bath. The mixture was slowly added to acetic acid (4 ml, 70 mmol) and stirred for 14 h. To the reaction mixture, saturated NaHCO_3_ aqueous solution (100 ml) was added to quench the reaction. Further, the reaction mixture was diluted with water and extracted with ethyl acetate (3 × 100 ml). The combined organic layers were dried over Na_2_SO_4_, filtered, and the solvents was removed under reduced pressure. The obtained residue was purified by silica gel column chromatography (hexane only to hexane/ethyl acetate = 3/1) to yield dimethylamine **3c** (912 mg, 2.96 mmol, 35%) as a white solid: ^1^H NMR (500 MHz, CDCl_3_) *δ* 8.50 (s, 1H), 8.31 (d, *J* = 8.6 Hz, 1H), 8.08 (dd, *J* = 9.2, 1.7 Hz, 1H), 7.86 (d, *J* = 8.6 Hz, 1H), 7.41 (d, *J* = 8.6 Hz, 1H), 3.97 (s, 3H), 2.95 (s, 6H); ^13^C NMR (126 MHz, CDCl_3_) *δ* 167.11, 152.03, 135.86, 131.09, 130.02, 129.85, 127.15, 126.82, 126.28, 120.87, 116.27, 52.32, 44.40; HR‐ESI‐MS: *m/z*: [M + H]^+^ calcd for C_14_H_15_BrNO_2_, 308.0286, 310.0266; found, 308.0295, 310.0274.

#### 2.2.3 Allyl dimethylamine **3d**

To a solution of dimethylamine **3c** (2.61 g, 8.46 mmol) in dimethylformamide (40 ml), allyltributylitin (3.4 ml, 11 mmol), LiCl (1.14 g, 28.3 mmol), and Pd (PPh_3_)_2_Cl_2_ (584 mg, 0.832 mmol) were added, and the mixture was stirred for 10 h at 90°C. The reaction mixture was purified by silica gel column chromatography with 10 wt% K_2_CO_3_ (hexane/ethyl acetate = 1/1). The obtained crude compound was purified by silica gel column chromatography (hexane/ethyl acetate = 4/1) to yield allyl dimethylamine **3d** (2.15 g, 7.99 mmol, 94%) as a colorless oil: ^1^H NMR (500 MHz, CDCl_3_) *δ* 8.54 (d, *J* = 1.7 Hz, 1H), 8.02 (dd, *J* = 8.9, 2.0 Hz, 1H), 7.95 (d, *J* = 8.6 Hz, 1H), 7.83 (d, *J* = 8.6 Hz, 1H), 7.45 (d, *J* = 8.6 Hz, 1H), 6.12–6.19 (m, 1H), 5.91–6.00 (m, 1H), 5.08 (dd, *J* = 10.3, 1.7 Hz, 1H), 4.89 (dd, *J* = 17.2, 1.7 Hz, 1H), 4.80 (dd, *J* = 16.6, 1.7 Hz, 1H), 4.66 (dd, *J* = 10.0, 2.0 Hz, 1H), 4.00 (q, *J* = 2.3 Hz, 1H), 3.96 (s, 3H), 2.79 (s, 6H); ^13^C NMR (126 MHz, CDCl_3_) *δ* 167.11, 152.03, 135.86, 131.09, 130.02, 129.85, 127.15, 126.82, 126.28, 120.87, 116.27, 52.32, 44.40; HR‐ESI‐MS: *m/z*: [M + H]^+^ calcd for C_17_H_20_NO_2_, 270.1494; found, 270.1448.

#### 2.2.4 Allyl alcohol **3e**

A solution of allyl dimethylamine **3d** (2.15 mg, 7.99 mmol) in dry toluene (30 ml) under Ar at 0°C was slowly added 1.0‐M diisobutylaluminium hydride (DIBAL‐H) in toluene (16.0 ml, 16 mmol), and the mixture was stirred for 1 h at r.t. Then to the reaction mixture was added 1‐M hydrochloric acid (10 ml). The mixed solution was extracted with ethyl acetate (3 × 100 ml). The combined organic layers were dried over Na_2_SO_4_, filtered, and the solvent was removed under reduced pressure. The obtained residue was purified by silica gel column chromatography (hexane/ethyl acetate = 3/1) to yield alcohol **3e** (1.65 mg, 6.83 mmol, 85%) as a colorless oil: ^1^H NMR (500 MHz, CDCl_3_) *δ* 7.92 (d, *J* = 9.2 Hz, 1H), 7.69 (d, *J* = 9.2 Hz, 2H), 7.41 (d, *J* = 8.6 Hz, 2H), 6.12 (qd, *J* = 11.0, 5.3 Hz, 1H), 5.03 (d, *J* = 10.3 Hz, 1H), 4.88 (d, *J* = 17.2 Hz, 1H), 4.78 (d, *J* = 6.9 Hz, 2H), 3.99 (q, *J* = 1.7 Hz, 2H), 2.75 (s, 6H); ^13^C NMR (126 MHz, CDCl_3_) *δ* 150.19, 137.84, 136.63, 132.92, 130.89, 128.76, 127.75, 126.04, 125.50, 125.31, 120.16, 115.60, 65.46, 45.77, 31.15; HR‐ESI‐MS: *m/z*: [M + H]^+^ calcd for C_16_H_20_NO, 242.1545; found, 242.1507.

#### 2.2.5 Allyl aldehyde **3f**

To a solution of alcohol **3e** (1.46 mg, 6.07 mmol) in dichloromethane (50 ml), Dess–Martin periodinane (2.71 g, 6.39 mmol) and pyridine (1.0 ml, 12 mmol) were added, and the mixture was stirred for 5 h at r.t. The reaction mixture was diluted with water and extracted with chloroform (3 × 100 ml). The combined organic layer was dried over Na_2_SO_4_, filtered, and the solvent was removed under reduced pressure. The obtained residue was purified by silica gel column chromatography (hexane/ethyl acetate = 5/1) to yield allyl aldehyde **3f** (477 mg, 2.00 mmol, 33%) as a yellow oil: ^1^H NMR (500 MHz, CDCl_3_) *δ* 10.11 (s, 1H), 8.26 (d, *J* = 1.7 Hz, 1H), 7.99 (d, *J* = 9.2 Hz, 1H), 7.87–7.90 (complex, 2H), 7.48 (d, *J* = 8.6 Hz, 1H), 6.12–6.20 (m, 1H), 5.09 (dq, *J* = 10.3, 1.9 Hz, 1H), 4.88 (dq, *J* = 17.2, 1.9 Hz, 1H), 3.97–3.99 (m, 2H), 2.82 (s, 6H); ^13^C NMR (126 MHz, CDCl_3_) *δ* 192.17, 153.35, 137.19, 136.84, 134.88, 132.27, 129.50, 129.36, 127.84, 125.83, 122.77, 120.70, 115.99, 45.18, 31.37; HR‐ESI‐MS: *m/z*: [M + H]^+^ calcd for C_16_H_18_NO, 240.1388; found, 240.1359.

#### 2.2.6 Allyl ethyl ester **3g**

A solution of allyl aldehyde **3f** (474 mg, 1.98 mmol) in toluene (15 ml) and (carbethoxymethylene)triphenylphosphorane (2.03 g, 5.82 mmol) was added, and the mixture was stirred for 7 h at r.t. The reaction mixture was purified by silica gel column chromatography (hexane/ethyl acetate = 7/1) to yield allyl ethyl ester **3g** (584 mg, 1.89 mmol, 95%) as a green‐yellow oil: ^1^H NMR (500 MHz, CDCl_3_) *δ* 7.91 (d, *J* = 9.2 Hz, 1H), 7.86 (d, *J* = 1.1 Hz, 1H), 7.82 (d, *J* = 16.0 Hz, 1H), 7.74 (d, *J* = 8.6 Hz, 1H), 7.63 (dd, *J* = 9.2, 1.7 Hz, 1H), 7.43 (d, *J* = 9.2 Hz, 1H), 6.50 (d, *J* = 16.0 Hz, 1H), 6.10–6.18 (m, 1H), 5.06 (dd, *J* = 10.3, 1.7 Hz, 1H), 4.89 (dd, *J* = 17.2, 1.7 Hz, 1H), 4.28 (q, *J* = 7.1 Hz, 2H), 3.98 (t, *J* = 2.6 Hz, 2H), 2.77 (s, 6H), 1.35 (t, *J* = 7.2 Hz, 3H); ^13^C NMR (126 MHz, CDCl_3_) *δ* 167.35, 151.61, 144.85, 137.58, 134.40, 130.55, 130.43, 130.17, 128.44, 125.71, 123.52, 120.56, 117.57, 115.80, 60.54, 45.51, 31.22, 14.46; HR‐ESI‐MS: *m/z*: [M + H]^+^ calcd for C_20_H_24_NO_2_, 310.1807; found, 310.1801.

#### 2.2.7 Carboxylic acid **3h**

A solution of allyl ethyl ester **3g** (198 mg, 0.642 mmol) in 2‐propanol (4 ml) was added 1‐M NaOH *aq.* (2 ml), and the mixture was heated at reflux for 3 h. After cooling, the reaction mixture was neutralized by adding 1‐M HCl *aq.* The mixed solution was extracted with ethyl acetate (3 × 100 ml). The combined organic layers were dried over Na_2_SO_4_, filtered, and the solvent was removed under reduced pressure to give carboxylic acid **3h** (148 mg, 0.525 mmol, 82%) as a green‐yellow solid: ^1^H NMR (500 MHz, CDCl_3_) *δ* 7.89–7.95 (m, 3H), 7.77 (d, *J* = 8.6 Hz, 1H), 7.66 (dd, *J* = 8.6, 1.7 Hz, 1H), 7.45 (d, *J* = 8.6 Hz, 1H), 7.26 (s, 2H), 6.53 (d, *J* = 16.0 Hz, 1H), 6.11–6.19 (m, 1H), 5.06–5.09 (m, 1H), 4.88–4.92 (m, 1H), 3.98 (t, *J* = 2.6 Hz, 2H), 2.78 (d, *J* = 7.4 Hz, 6H); ^13^C NMR (126 MHz, CDCl_3_) *δ* 172.26, 151.76, 147.09, 137.44, 134.59, 130.91, 130.39, 129.70, 128.50, 128.27, 125.73, 123.49, 120.53, 116.54, 115.77, 45.39, 31.16; HR‐ESI‐MS: *m/z*: [M + H]^+^ calcd for C_18_H_20_NO_2_, 282.1494; found, 282.1469.

#### 2.2.8 Amide **3i**

To a solution of carboxylic acid **3h** (255 mg, 0.905 mmol) and *S*‐trityl‐*d*‐cysteine methyl ester (521 mg, 1.38 mmol) in dimethylformamide (10 ml), 4‐(4,6‐dimethoxy‐1,3,5‐triazin‐2‐yl)‐4‐methylmorpholinium chloride (DMT‐MM) was added, and the reaction mixture was stirred for 7 h at r.t. The reaction was quenched by adding water (50 ml), and the product was extracted with ethyl acetate (3 × 100 ml). The combined organic layer was dried over Na_2_SO_4_, filtered, and concentrated under reduced pressure. The residue was purified by silica gel column chromatography (hexane/ethyl acetate = 2/1) to yield amide **3i** (551 mg, 0.859 mmol, 95%) as a green‐yellow solid ^1^H NMR (500 MHz, CDCl_3_) *δ* 7.93 (d, *J* = 8.6 Hz, 1H), 7.85 (d, *J* = 1.7 Hz, 1H), 7.75 (s, 1H), 7.73 (d, *J* = 6.9 Hz, 1H), 7.62 (dd, *J* = 9.2, 1.7 Hz, 1H), 7.44 (d, *J* = 9.2 Hz, 1H), 7.39–7.41 (m, 6H), 7.27–7.30 (m, 6H), 7.19–7.24 (m, 3H), 6.44 (d, *J* = 15.5 Hz, 1H), 6.11–6.19 (m, 2H), 5.06–5.09 (m, 1H), 4.90–4.94 (m, 1H), 4.78–4.82 (m, 1H), 3.99 (q, *J* = 1.9 Hz, 2H), 3.74 (s, 3H), 2.78 (d, *J* = 4.0 Hz, 6H); ^13^C NMR (126 MHz, CDCl_3_) *δ* 171.01, 165.45, 151.34, 144.31, 141.98, 137.51, 134.13, 130.56, 130.24, 130.03, 129.50, 128.40, 128.30, 128.03, 126.92, 125.57, 123.43, 120.43, 119.20, 115.70, 66.98, 52.71, 51.20, 45.45, 34.02, 31.11,; HR‐ESI‐MS: *m/z*: [M + Na]^+^ calculated for C_41_H_40_N_2_NaO_3_S, 663.2657; found, 663.2703, [M + K]^+^ calcd for C_41_H_40_N_2_KO_3_S, 679.2397; found, 679.2415.

#### 2.2.9 Thiazolidine ester **3j**

To a solution of trifluoromethanesulfonic anhydride (Tf_2_O) (0.30 ml, 1.8 mmol) in dichloromethane (5 ml), a solution of amide **3i** (540 mg, 0.843 mmol) in dichloromethane (5 ml) was added under Ar at 0°C, and the mixture was stirred for 10 min. Saturated NaHCO_3_ *aq.* was added to the reaction mixture for neutralization. The product was extracted with chloroform (3 × 50 ml). The combined organic layer was dried over Na_2_SO_4_, filtered, and concentrated under reduced pressure. The crude products were purified by silica gel column chromatography (hexane/ethyl acetate = 5/1) to yield thiazolidine ester **3j** (122 mg, 0.322 mmol, 39%) as orange oil: ^1^H NMR (500 MHz, CDCl_3_) *δ* 7.91 (d, *J* = 9.2 Hz, 1H), 7.81 (d, *J* = 1.7 Hz, 1H), 7.73 (d, *J* = 8.6 Hz, 1H), 7.62 (dd, *J* = 9.2, 1.7 Hz, 1H), 7.43 (d, *J* = 8.6 Hz, 1H), 7.28 (d, *J* = 16.0 Hz, 1H), 7.17 (d, *J* = 16.0 Hz, 1H), 6.11–6.16 (m, 1H), 5.22 (t, *J* = 9.2 Hz, 1H), 5.07 (dd, *J* = 10.3, 1.7 Hz, 1H), 4.90 (dd, *J* = 17.2, 1.7 Hz, 1H), 3.97–3.98 (m, 2H), 3.84 (s, 3H), 3.65 (dd, *J* = 10.9, 9.2 Hz, 1H), 3.58 (dd, *J* = 10.9, 9.2 Hz, 1H), 2.77 (s, 6H); ^13^C NMR (126 MHz, CDCl_3_) *δ* 171.31, 170.32, 151.37, 142.51, 137.48, 134.04, 130.52, 129.68, 128.41, 128.23, 125.71, 123.09, 121.60, 120.45, 115.72, 77.95, 52.87, 45.44, 34.61, 31.10; HR‐ESI‐MS: *m/z*: [M + H]^+^ calcd for C_22_H_25_N_2_O_2_S, 381.1637; found, 381.1623.

#### 2.2.10 NIR analogue **3**

A solution of thiazolidine ester **3j** (32.8 mg, 0.0862 mmol) in 4 M HCl *aq.* (1 ml) and tetrahydrofuran (1 ml) was stirred at r.t. for 18 h. After neutralization of the reaction mixture by adding NaHCO_3_, the mixture was then concentrated under reduced pressure. The crude products were purified by automated flash chromatography (Smart Flash EPCLC AI‐580S, ULTRAPACK COLUMNS C18, H_2_O/methanol = 9/1 to 1/9) to yield NIR analogue **3** (8.7 mg, 0.023 mmol, 28%) as an orange solid: m.p. 160–164 °C; IR (attenuated total reflection, cm^−1^): 1590, 1369, 1195, 1143, 983, 955, 816; ^1^H NMR (500 MHz, CD_3_OD) *δ* 7.91 (t, *J* = 9.5 Hz, 2H), 7.77 (d, *J* = 9.2 Hz, 1H), 7.67 (dd, *J* = 8.9, 1.4 Hz, 1H), 7.47 (d, *J* = 8.6 Hz, 1H), 7.36 (d, *J* = 16.0 Hz, 1H), 7.15 (d, *J* = 16.0 Hz, 1H), 6.08–6.15 (m, 1H), 5.17 (t, *J* = 8.9 Hz, 1H), 5.02 (dd, *J* = 10.3, 1.7 Hz, 1H), 4.80–4.84 (m, 1H), 3.96 (t, *J* = 2.6 Hz, 2H), 3.59–3.69 (m, 2H), 2.75 (s, 6H); ^13^C NMR (126 MHz, CD_3_OD) *δ* 173.25, 171.83, 151.38, 143.03, 137.56, 134.08, 130.89, 130.68, 129.59, 128.38, 128.32, 125.53, 122.90, 120.44, 120.24, 114.61, 77.63, 44.51, 34.34, 30.59; HR‐ESI‐MS: *m/z*: [M + H]^+^ calcd for C_21_H_23_N_2_O_2_S, 367.1480; found, 367.1437; 93%e.e. from chiral HPLC on a CHIRALCEL OD‐RH (retention time of *l*‐isomer: 13.69 min; *d*‐isomer: 14.43 min; H_2_O containing with 0.1% formic acid/acetonitrile = 90/10 to 10/90; UV detection: 254 nm).

### 2.3 Luminescence measurements

Bioluminescence activities of **3** together with those of wild‐type luciferin **1** and TokeOni (**2**) were investigated using *Ppy* luciferase and Akaluc. The substrates were dissolved in 50‐mM potassium phosphate buffer (KPB, pH 6.0), *Ppy* luciferase and Akaluc were dissolved in 50‐mM KPB (pH 8.0) containing 35% glycerol, and Mg‐ATP was dissolved in ultrapure water. An L–L reaction was initiated by injection of 10 μl of Mg‐ATP (200 μM) into a mixture of 5 μl of a substrate solution (100 μM), 5 μl of luciferase solution (1 mg/ml), and 5 μl of KPB (500 mM, pH 8.0). Emission spectra were measured on the AB‐1850 spectrophotometer in the range of 400–790 nm (slit width: 1.0 mm; exposure time: 10 min [**3** and **5**] or 15 s [**1** and **2**]). Light emission intensity by *Ppy* luciferase was monitored on an AB‐1850 spectrophotometer to provide emission spectra (slit width: 1.0 mm; exposure time: 1 s; scan: 600), and light intensity was determined as the intensity at the *λ*_BL_ value of the emission spectrum.

Chemiluminescence emission spectra for the reactions of the luciferin methyl esters of **1**–**2**, **5**, and **3j** with *t*‐BuOK in DMSO under air were measured on an AB‐1850 spectrophotometer (slit width: 1.0 mm; exposure time: 180 s). A solution of the luciferin methyl ester (2.5 mm) in DMSO (200 μl) was placed in a polystyrene tube. This solution was mixed with *t*‐BuOK (250 mm) in DMSO (200 μl), which was injected with a syringe, to initiate the chemiluminescence reaction with final concentrations of the substrate (1.25 mm) and *t*‐BuOK (125 mm).

## 3 RESULTS AND DISCUSSION

### 3.1 Synthesis of luciferin analogue 3

**SCHEME 1:**
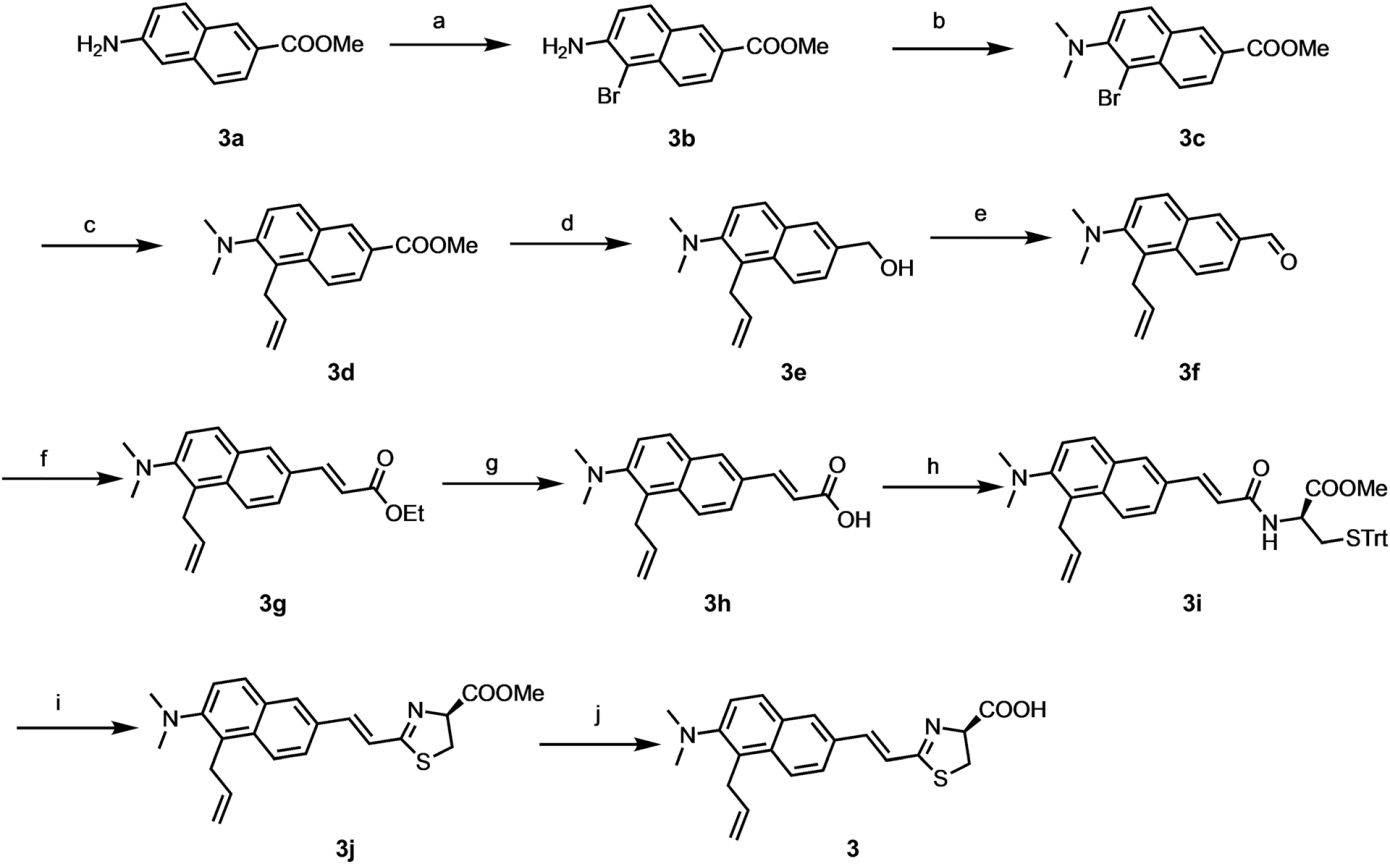
Synthetic routes for luciferin analogue **3**. A, NBS, DMSO, r.t.; B, NaBH_3_CN, formaldehyde, CH_3_COOH, CH_3_OH, 0°C to r.t.; C, Allyltributyltin, Pd (PPh_3_)_2_Cl_2_, LiCl, DMF, 90°C; D, DIBAL‐H, toluene, 0°C to r.t.; E, DMP, pyridine, CH_2_Cl_2_, 0°C to r.t.; F, Ph_3_PCHCOOEt, toluene, r.t.; G, NaOH *aq*., *i*PrOH, reflux; H, *d*‐Cys (Trt)‐OMe, DMT‐MM, DMF, r.t.; I, Tf_2_O, CH_2_Cl_2_, 0 °C; J, HCl *aq*., THF, r.t

Analogue **3** was prepared according the procedure as shown in Scheme 1. The synthesis of **3** was started from bromination of commercially available methyl ester **3a** to obtain **3b**. Dimethylation of **3b** followed by allylation yielded **3d**. Alcohol **3e** was prepared from **3d** via diisobutylaluminium hydride (DIBAL‐H) reduction. Allylaldehyde **3f** was prepared via oxidation of **3e**, followed by Dess–Martin periodinane. Wittig reaction of **3f** with (carbethoxymethylene)triphenylphosphorane (Ph_3_PCHCOOEt) afforded ethyl ester **3g**, which was hydrolyzed to give carboxylic acid **3h**. The condensation of **3h** with *S*‐trityl‐*d*‐cysteine methyl ester gave amide **3i**, and the following thiazoline ring formation with trifluoromethanesulfonic anhydride (Tf_2_O) and triphenylphosphine oxide (Ph_3_PO) afforded ester **3j**. Finally, acid hydrolysis of **3j** produced target analogue **3**.

### 3.2 Bioluminescence activity of analogues **3** and **5**

**FIGURE 3:**
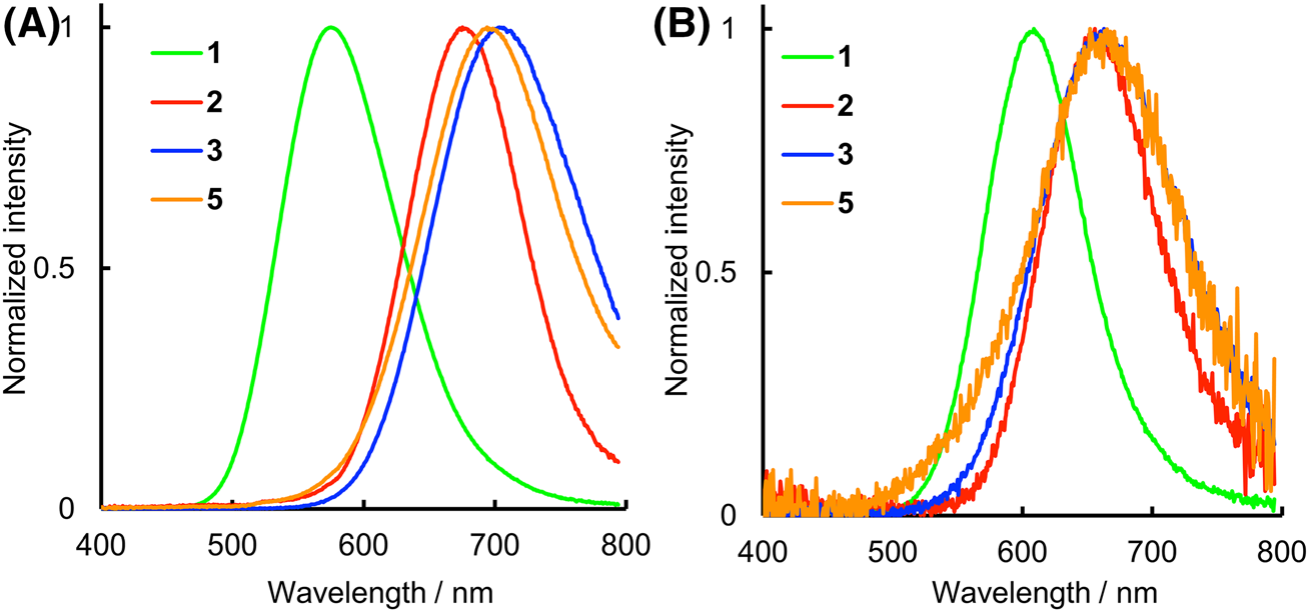
The bioluminescence spectra of **1**–**3** and **5** reacted with *Ppy* luciferase A, and Akaluc B, respectively

BL activity and emission spectrum of **3** together with those of **1**, **2**, and **5** were investigated with wild‐type recombinant *Ppy* luciferase and a mutant luciferase, Akaluc (Table 1 and Figure 3).

**TABLE 1:** Bioluminescence and chemiluminescence properties of **1**–**3** and **5**

Compound	Rel. Int.^a^ (*Ppy* luc.)	*λ*_BL_ ^b^/nm (*Ppy* luc.)	*λ*_BL_ ^c^/nm (Akaluc)	*λ*_CL_ ^d^/nm
**1**	100%	570	610	595
**2**	10%	675	660	685
**3**	1.3%	705	665	685
**5**	0.8%	690	660	620

a Relative light intensity at *λ*_BL_ upon reaction with *Ppy* luciferase for the L–L reactions of **3** during the initial 600 s compared with that of **1**.

b Bioluminescence emission maximum upon reaction with *Ppy* luciferase.

c Bioluminescence emission maximum upon reaction with mutant luciferase Akaluc.

d Chemiluminescence emission maximum.

Before investigating BL properties, the *d*‐ and *l*‐forms of **3** were separated by HPLC with a chiral octadecylsilane column, and their fractions were screened for BL measurements. The *d*‐form of **3** showed sufficient luminescence with *Ppy* luciferase, whereas the *l*‐form of **3** showed negligible luminescence similar to the background (Table S1). Similar to wild‐type luciferin **1**, NIR luciferin analogue produces light only in the *d*‐form and not in the *l*‐form. We used only *d*‐form of **3** for the following experiments. The light intensity (Rel. Int.) obtained through the L–L reaction with *Ppy* luciferase during the initial 600 s for **3** was 1.3% as a relative value compared with that for **1** (Table 1), and the Rel. Int. value was similar to that of **5** (0.8%). The light intensity of **3** with Akaluc was weaker than that with *Ppy* luciferase and could not be determined relative intensity. These results indicate that **3** and **5** have weak BL activities compared with **2**. The *λ*_BL_ value of **3** was recorded at 705 nm with *Ppy* luciferase (Table 1 and Figure 3), which red shifted from that of **5** (690 nm). The *λ*_BL_ values of **3** and **5** are 135 and 120 nm longer than that of **1**, respectively, and even 30 and 15 nm longer than that of **2**, respectively. On the other hand, the emission spectra of **3** measured with Akaluc showed the *λ*_BL_ value at 665 nm (Table 1 and Figure 3), which is red shifted by 15 nm compared with that of **2**. Also, the *λ*_BL_ value of **5** reacted with Akaluc was observed at 660 nm that is same as that of **2**. To investigate the cause of the variation in *λ*_BL_ values for **3** and **5**, chemiluminescence reaction of the methyl esters of **1**–**3** and **5** were performed in DMSO containing *t‐*BuOK under air. The chemiluminescence emission maxima (*λ*_CL_) of **1**–**3** and **5** were observed at 595, 685, 685, and 620 nm, respectively (Table 1 and Figure S1). The *λ*_CL_ value of **3** is same as that of **2**, and the *λ*_CL_ value of **5** is blue shifted by 65 nm compared with that of **2**.

## 4 DFT AND TIME‐DEPENDENT DFT CALCULATIONS FOR *OXY*‐**2**, *OXY*‐**3**, AND *OXY*‐**5**

**FIGURE 4:**
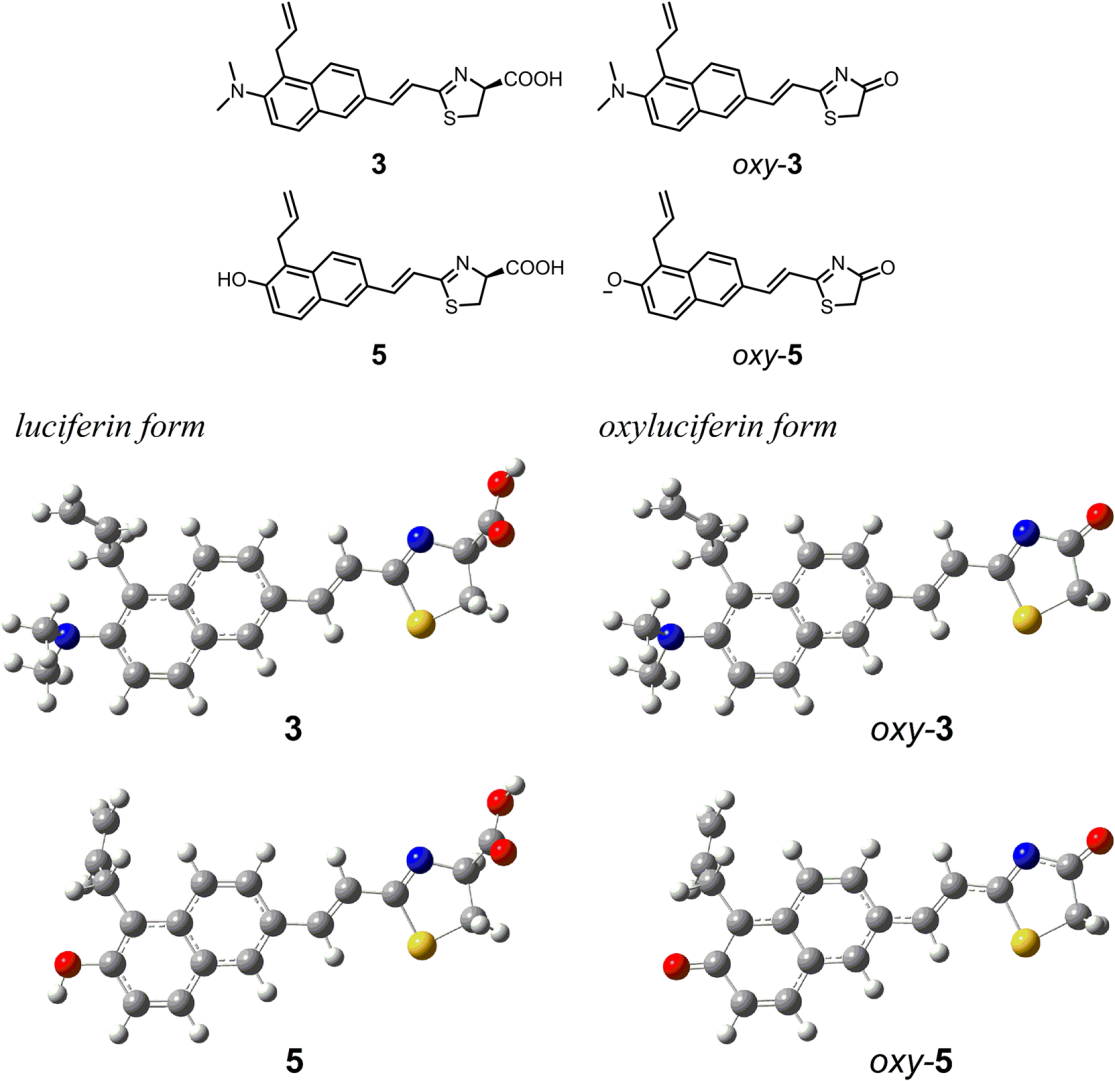
The most stable optimized structures of the luciferin forms **3** and **5** and optimized structures of the oxyluciferin forms *oxy*‐**3** and *oxy*‐**5**(phenolate) having the conformations corresponding to the structures of **3** and **5**

To further evaluate the observed *λ*_BL_ and *λ*_CL_ values for **3**, the electronic properties of the oxyluciferin form of **3** (*oxy*‐**3**) together with that of the oxyluciferin form of **5** (*oxy*‐**5**) were investigated using DFT and time‐dependent DFT (TD‐DFT) calculations with the B3LYP/6–31 + G(d) method. Prior performing a search for the most stable optimized structures of *oxy*‐**3** and *oxy*‐**5**, we found the most stable optimized structures of the luciferin forms **3** and **5**. We then used the structures of **3** and **5** shown in Figure 4 as the basis for starting conformations of *oxy*‐**3** and *oxy*‐**5** for further calculations because the structures of **3** and *oxy*‐**3** have steric hindrance between the allyl and dimethylamino groups, and their dimethylamino groups are twisted and pyramidal. Next, we analyzed the electronic transition properties of the oxyluciferin forms (Table 2). In the case of *oxy*‐**5**, the phenolate anion and its sodium salt model were calculated in the manner similar to the previous literature.^2^

**TABLE 2:** Time‐dependent density functional theory calculation data for *oxy*‐**2**, *oxy*‐**3**, and *oxy*‐**5**

Compound	Transition^a^	*E*_ex_/eV^b^	*λ*_ex_/nm (*f*)^c^	Configuration^d^
*oxy‐* **2** ^e^	S_0_ → S_1_	2.82	439 (1.38)	H → L (0.70)
*oxy‐* **3**	S_0_ → S_1_	2.85	435 (0.58)	H → L (0.70)
*oxy*‐**5**(phenolate)	S_0_ → S_1_	2.40	516 (1.23)	H → L (0.71) H ← L (−0.14)
*oxy*‐**5**(ONa)	S_0_ → S_2_	2.67	464 (0.87)	H → L + 1 (0.70)

a The allowed transition to the excited singlet state with the lowest excitation energy (S_0_ → S_1_ or S_0_ → S_2_).

b Vertical excitation energy for the transition.

c Wavelength (*λ*_ex_) estimated from the transition energy. Oscillator strength (*f*) is in the parenthesis.

d Configuration of excitation. Coefficient is in the parenthesis. H, L, and L + 1 denote highest occupied molecular orbital (HOMO), lowest unoccupied molecular orbital (LUMO), and LUMO+1, respectively.

e Kiyama et al.^16^

Table 2 summarizes vertical excitation energies (*E*_ex_), excitation wavelengths (*λ*_ex_), oscillator strengths (*f*), and configurations of the allowed transitions to the excited singlet states with the lowest energies for *oxy*‐**3**, *oxy*‐**5**(phenolate), and *oxy*‐**5**(ONa) together with those for *oxy*‐**2**.^16^ The S_0_ → S_1_ transitions of *oxy*‐**3** and *oxy*‐**5**(phenolate) are π, π* transitions corresponding to the highest occupied molecular orbital (HOMO) → lowest unoccupied molecular orbital (LUMO) configuration and the S_0_ → S_2_ transition of *oxy*‐**5**(ONa) is a π, π* transition corresponding to the HOMO → LUMO + 1 configuration. Although the *λ*_BL_ value of **3** with *Ppy* luciferase is red shifted from that of **2**, the *λ*_ex_ values of *oxy*‐**2** and *oxy*‐**3** are similar. Results indicate that *λ*_BL_ values were mainly determined by the effect of the active site of *Ppy* luciferase to stabilize the excited states of *oxy*‐**2** and *oxy*‐**3**. Because the HOMO–LUMO transition of *oxy*‐**2** has charge‐transfer character, the S_1_ state is more highly polarized than the ground state.^20^ The HOMO and LUMO of *oxy*‐**3** have primary electronic distributions at the (6‐dimethylaminonaphtalenyl) and 2‐ethenyl‐1,3‐thiazolone moieties, respectively (Figure 4), indicating that the HOMO–LUMO transition of *oxy*‐**3** also has charge‐transfer character. Thus, the S_1_ state of *oxy*‐**3** also has polarized character. The environment surrounding the excited *oxy*‐**3** in *Ppy* luciferase will be more polar than that surrounding the excited *oxy*‐**2**, resulting in the red‐shifted *λ*_BL_ value of **3**. The electronic distributions of the HOMO and LUMO of *oxy*‐**3** indicate that the allyl group has no contribution to the π electronic conjugation. The calculations showing that the *λ*_ex_ values of *oxy*‐**5**(phenolate) and *oxy*‐**5**(ONa) are red shifted from that of *oxy*‐**3** are opposite to the *λ*_BL_ data with *Ppy* luciferase and Akaluc. Although the oxido (O^−^) group of *oxy*‐**5**(phenolate) and *oxy*‐**5**(ONa) has the potential to donate more electron density than that of the dimethylamiono group of *oxy*‐**3**, the anionic property of the oxido group in the luciferase active site may be weakened.

## 5 CONCLUSION

We synthesized luciferin analogue **3** and investigated their luminescence properties. The *λ*_BL_ values for **3** upon reaction with *Ppy* luciferase and mutant luciferase Akaluc were 705 and 665 nm, respectively. Furthermore, the results of BL and TD‐DFT calculations suggest that the allyl group of **3** induced the excited *oxy*‐**3** to be more stable in the active site of luciferase, thus increasing the *λ*_BL_ value of **3** to over 700 nm. A *λ*_BL_ value of over 700 nm is quite noteworthy; however, the intensity of **3** was very weak compared with those of **1** and **2**. We should modify the new analogue design to produce a higher bioluminescence intensity for animal experiments.

## Supporting information

Data S1 Supporting information
