# Supplementary material for: Development of near‐infrared firefly luciferin analogue reacted with wild‐type and mutant luciferases
Source: Chirality. 2020 May 4;32(7):922–31. doi: 10.1002/chir.23236 (PMC7383472; doi:10.1002/chir.23236)
Supplement: Supplementary file 1 — Data S1 Supporting information [file CHIR-32-922-s001.docx]

**Supporting Information**

**Tittle**

Development of near-infrared firefly luciferin analogue reacted with wild type and mutant luciferases

Nobuo Kitada^1,2†^, Ryohei Saito^1,3†^, Rika Obata^1^, Satoshi Iwano^4^, Kazuma Karube^1^, Atsushi Miyawaki^4^, Takashi Hirano^1^ and Shojiro A. Maki^1,2^*

^†^These authors contributed equally to this work.

*Correspondence and requests for materials should be addressed to S.A.M. (email: [s-maki@uec.ac.jp](mailto:s-maki@uec.ac.jp)).

1 Department of Engineering Science, Graduate School of Informatics and Engineering, The University of Electro-Communications, 1-5-1 Chofugaoka, Chofu, Tokyo 182-8585, Japan

2 Center for Neuroscience and Biomedical Engineering, The University of Electro-Communications, 1-5-1 Chofugaoka, Chofu, Tokyo 182-8585, Japan

3 (Current) School of Pharmacy, Tokyo University of Pharmacy and Life Science, 1432-1 Horinouchi, Hachioji, Tokyo 192-0392, Japan

4 Laboratory for Cell Function and Dynamics, Center for Brain Science, RIKEN, 2-1 Hirosawa, Wako, Saitama 351-0198, Japan

**S1. Luminescence measurements S16**

**S2. DFT calculations S18**

**S1. Luminescence measurements**

Bioluminescence activity of *d*-**3** and *l*-**3**

*d*-form of **3** (*d*-**3**) and *l*-form of **3** (*l*-**3**) fractions were collected on a chiral column and measured for luminescence. *d*-**3** and *l*-**3** were separated by chiral HPLC on a Chiralcel OD-RH (retention time of *l*-form: 13.69 min, *d*-form: 14.43 min, H_2_O containing with 0.1% formic acid/acetonitrile = 90/10 to 10/90). And each fraction was identified by HR-MS (HR-ESI-MS: *d*-**3** m/z: [M+H]^+^ calculated for C_21_H_23_N_2_O_2_S, 367.1480; found, 367.1439; *l*-**3** m/z: [M+H]^+^ calculated for C_21_H_23_N_2_O_2_S, 367.1480; found, 367.1457).

The bioluminescence activities of *d*-**3** and *l*-**3** were investigated using *Ppy* luciferase. HPLC fractions of the substrates were used directly, *Ppy* luciferase was dissolved in 50 mM KPB (pH 8.0) containing 35% glycerol, and Mg-ATP was dissolved in ultrapure water. An L-L reaction was initiated by injection of 40 μL of Mg-ATP (10 mM) into a mixture of 20 μL of a substrate solution, 20 μL of luciferase solution (0.01 mg/mL), and 20 μL of KPB (500mM, pH 8.0). The emission intensity was measured on an AB-2270(exposure time: 30 sec).

**Table S1** Luminescence intensity (photon count) of *d*-**3** and *l*-**3**

| Compound | *d*-**3** | *l*-**3** | background |
| --- | --- | --- | --- |
| Intensity | 1.54 × 10^5^ | 2.80 × 10^3^ | 2.68 × 10^3^ |

Spectra of chemiluminescence


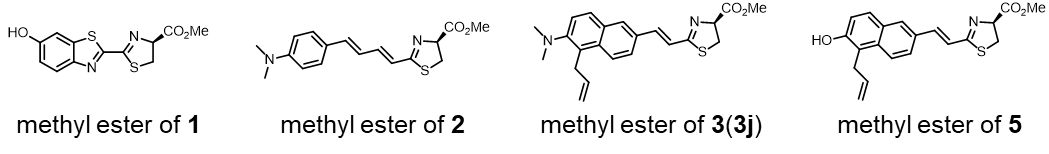

**Figure S1** Emission spectra of chemiluminescence for luciferin methyl ester of **1**–**2,** **5** and **3j**

**S2. DFT Calculation**

2.1. Heats of formation

**Table S2.** Heats of formation of luciferin analogues **3** and **5** and the corresponding oxyluciferins *oxy*-**3**, *oxy*-**5**(phenolate), and *oxy*-**5**(ONa) in gas phase optimized with DFT using B3LYP/6-31+G(d).^1,2,3,4,5^

| Compound | Heat of formation/hartree |
| --- | --- |
| **3** | −1471.61223394 |
| *oxy*-**3** | −1357.07253194 |
| **5** | −1412.87116392 |
| *Oxy-***5**(phenolate) | −1297.81856256 |
| *oxy*-**5**(ONa) | −1460.06954660 |

2.2. Geometries of luciferin analogues **3** and **5** and the corresponding oxyluciferins *oxy*-**3**, *oxy*-**5**(phenolate), and *oxy*-**5**(ONa) in gas phase optimized with DFT using B3LYP/6-31+G(d)

**Table S3.** Cartesian coordinates (in Å) of luciferin analogue **3**

| No | atom | x | y | z |
| --- | --- | --- | --- | --- |
| 1 | C | 3.0318882 | 0.8603359 | -0.0580626 |
| 2 | S | 2.4372738 | 2.5316643 | 0.2791956 |
| 3 | N | 4.3048584 | 0.6968131 | -0.1224762 |
| 4 | C | 4.1301449 | 3.1584739 | -0.049523 |
| 5 | H | 4.1939096 | 3.5288187 | -1.0756518 |
| 6 | H | 4.3663564 | 3.967325 | 0.6454911 |
| 7 | C | 2.1077828 | -0.2538978 | -0.2172584 |
| 8 | C | 0.7609378 | -0.141119 | -0.2202305 |
| 9 | H | 2.6028631 | -1.2131755 | -0.3406727 |
| 10 | H | 0.326271 | 0.8523434 | -0.1110374 |
| 11 | C | -0.2135865 | -1.2197839 | -0.3667766 |
| 12 | C | -1.567043 | -0.9098607 | -0.390281 |
| 13 | H | -1.8807557 | 0.1281697 | -0.2930612 |
| 14 | C | -2.5691523 | -1.9012688 | -0.5365402 |
| 15 | C | -3.9468958 | -1.5635283 | -0.5380345 |
| 16 | C | -2.1950813 | -3.2798015 | -0.6737917 |
| 17 | C | -4.9031605 | -2.540477 | -0.6770313 |
| 18 | H | -4.2378934 | -0.5204754 | -0.4342465 |
| 19 | C | -3.2005216 | -4.2893212 | -0.8168247 |
| 20 | C | -4.5493217 | -3.9121348 | -0.826473 |
| 21 | H | -5.9517628 | -2.2604795 | -0.6856271 |
| 22 | N | -5.570422 | -4.9024681 | -0.9673632 |
| 23 | C | -5.80986 | -5.3451295 | -2.343979 |
| 24 | H | -6.3424006 | -4.5790287 | -2.9381276 |
| 25 | H | -4.8661792 | -5.5729839 | -2.8416541 |
| 26 | H | -6.421957 | -6.2549344 | -2.3302781 |
| 27 | C | -6.8194541 | -4.6530006 | -0.2569719 |
| 28 | H | -7.4699931 | -3.9042135 | -0.7466091 |
| 29 | H | -7.383631 | -5.5913576 | -0.2025571 |
| 30 | H | -6.6099207 | -4.3206552 | 0.7638285 |
| 31 | C | -0.7966403 | -3.5774553 | -0.6315041 |
| 32 | H | -0.4629757 | -4.605902 | -0.7156175 |
| 33 | C | 0.152629 | -2.5949012 | -0.4843743 |
| 34 | H | 1.2006029 | -2.8777587 | -0.4559204 |
| 35 | C | 5.0444525 | 1.9301802 | 0.1496507 |
| 36 | H | 5.4070109 | 1.8721809 | 1.1860113 |
| 37 | C | 6.2695868 | 1.9823712 | -0.7665596 |
| 38 | O | 6.3306227 | 2.5673818 | -1.8264443 |
| 39 | O | 7.300795 | 1.2781608 | -0.2525998 |
| 40 | H | 8.0285505 | 1.3087737 | -0.9031467 |
| 41 | C | -2.8032199 | -5.7542836 | -0.9015194 |
| 42 | H | -3.6716427 | -6.3695349 | -0.6476989 |
| 43 | H | -2.0426025 | -5.9536778 | -0.1319105 |
| 44 | C | -2.2490098 | -6.1848278 | -2.2451548 |
| 45 | C | -2.5853572 | -7.3084022 | -2.8872896 |
| 46 | H | -1.510526 | -5.52254 | -2.6983696 |
| 47 | H | -2.1329393 | -7.5763139 | -3.8390468 |
| 48 | H | -3.3211474 | -8.0025382 | -2.4837036 |

**Table S4.** Cartesian coordinates (in Å) of oxyluciferin form, *oxy-***3**

| No | atom | x | y | z |
| --- | --- | --- | --- | --- |
| 1 | C | 3.0257583 | 0.859529 | -0.1555223 |
| 2 | S | 2.4248647 | 2.5439233 | 0.0307406 |
| 3 | N | 4.3129691 | 0.6752904 | -0.1593149 |
| 4 | C | 4.1486833 | 3.1187274 | 0.121169 |
| 5 | H | 4.3724339 | 3.8175672 | -0.6892345 |
| 6 | H | 4.3476827 | 3.6112586 | 1.0767419 |
| 7 | C | 2.1087201 | -0.2523348 | -0.2944792 |
| 8 | C | 0.7580815 | -0.1381297 | -0.3052627 |
| 9 | H | 2.6030785 | -1.2140742 | -0.3938223 |
| 10 | H | 0.3232435 | 0.8566281 | -0.2062979 |
| 11 | C | -0.2140553 | -1.2136883 | -0.4377934 |
| 12 | C | -1.5687437 | -0.900784 | -0.4479157 |
| 13 | H | -1.8794895 | 0.1382285 | -0.3522784 |
| 14 | C | -2.5723027 | -1.891141 | -0.5748128 |
| 15 | C | -3.9495341 | -1.5519756 | -0.5572454 |
| 16 | C | -2.201033 | -3.2715881 | -0.7083116 |
| 17 | C | -4.90804 | -2.5288287 | -0.6751866 |
| 18 | H | -4.2384775 | -0.5081531 | -0.4565396 |
| 19 | C | -3.2074671 | -4.2808751 | -0.8298177 |
| 20 | C | -4.5580744 | -3.9025005 | -0.8223083 |
| 21 | H | -5.9559529 | -2.2473292 | -0.6703353 |
| 22 | N | -5.5812966 | -4.8883852 | -0.9349879 |
| 23 | C | -5.8052512 | -5.4071092 | -2.2878803 |
| 24 | H | -6.3445559 | -4.6795135 | -2.9221616 |
| 25 | H | -4.8571304 | -5.6475106 | -2.7698701 |
| 26 | H | -6.4057583 | -6.3225946 | -2.2294248 |
| 27 | C | -6.838464 | -4.6114146 | -0.2496734 |
| 28 | H | -7.4831901 | -3.8848993 | -0.7783021 |
| 29 | H | -7.4013597 | -5.5479424 | -0.1640721 |
| 30 | H | -6.6420728 | -4.2379335 | 0.7592972 |
| 31 | C | -0.8014518 | -3.5711262 | -0.6815783 |
| 32 | H | -0.4704844 | -4.600307 | -0.764674 |
| 33 | C | 0.1508857 | -2.5902912 | -0.5526428 |
| 34 | H | 1.1987343 | -2.8738786 | -0.536999 |
| 35 | C | 5.0401925 | 1.8612651 | -0.0132798 |
| 36 | C | -2.8094033 | -5.7467431 | -0.897003 |
| 37 | H | -3.6731592 | -6.3605835 | -0.6244971 |
| 38 | H | -2.0433477 | -5.9321348 | -0.129314 |
| 39 | C | -2.2624129 | -6.199444 | -2.2368285 |
| 40 | C | -2.5824568 | -7.345906 | -2.8457348 |
| 41 | H | -1.5422866 | -5.5345613 | -2.7149349 |
| 42 | H | -2.1342558 | -7.6295484 | -3.7948168 |
| 43 | H | -3.2991548 | -8.0442982 | -2.416036 |
| 44 | O | 6.2521409 | 1.9327325 | 0.0098568 |

**Table S5.** Cartesian coordinates (in Å) of luciferin analogue **5**

| No | atom | x | y | z |
| --- | --- | --- | --- | --- |
| 1 | C | 3.0870352 | 0.9411471 | -0.1194608 |
| 2 | S | 2.4780557 | 2.6310432 | 0.0679164 |
| 3 | N | 4.3605087 | 0.7735744 | -0.0799456 |
| 4 | C | 4.1936449 | 3.2347278 | -0.1751693 |
| 5 | H | 4.3346113 | 3.5380637 | -1.2154914 |
| 6 | H | 4.3823447 | 4.0867209 | 0.4818178 |
| 7 | C | 2.1713999 | -0.1806479 | -0.2731308 |
| 8 | C | 0.8279549 | -0.0683324 | -0.3698121 |
| 9 | H | 2.669852 | -1.1456164 | -0.3027975 |
| 10 | H | 0.3916365 | 0.9302137 | -0.3486867 |
| 11 | C | -0.1404498 | -1.1531359 | -0.509473 |
| 12 | C | -1.4903964 | -0.8445165 | -0.6140212 |
| 13 | H | -1.8048686 | 0.1975982 | -0.5913324 |
| 14 | C | -2.4866743 | -1.8427758 | -0.7510736 |
| 15 | C | -3.8616297 | -1.5023533 | -0.8513871 |
| 16 | C | -2.1067619 | -3.2273752 | -0.7890705 |
| 17 | C | -4.8156384 | -2.4834796 | -0.9799005 |
| 18 | H | -4.1540263 | -0.4555203 | -0.8227036 |
| 19 | C | -3.102276 | -4.2412283 | -0.9391588 |
| 20 | C | -4.4328195 | -3.8471615 | -1.020583 |
| 21 | H | -5.8697755 | -2.2202145 | -1.0522639 |
| 22 | C | -0.714202 | -3.5237347 | -0.6704589 |
| 23 | H | -0.3876467 | -4.5573758 | -0.6689516 |
| 24 | C | 0.2279017 | -2.5328599 | -0.5372701 |
| 25 | H | 1.2731255 | -2.8122555 | -0.4452987 |
| 26 | C | 5.0839382 | 2.0212368 | 0.1695861 |
| 27 | H | 5.3660637 | 2.0286867 | 1.2322399 |
| 28 | C | 6.3753082 | 2.0149974 | -0.6519826 |
| 29 | O | 6.518671 | 2.5287795 | -1.7404594 |
| 30 | O | 7.3621513 | 1.3482497 | -0.0154963 |
| 31 | H | 8.1374647 | 1.3369445 | -0.6092551 |
| 32 | O | -5.3865581 | -4.8295607 | -1.1433095 |
| 33 | H | -6.2649942 | -4.4280734 | -1.2259527 |
| 34 | C | -2.7555457 | -5.719862 | -1.0016285 |
| 35 | H | -1.8307904 | -5.8744649 | -1.5689389 |
| 36 | H | -3.5512134 | -6.2323225 | -1.5551852 |
| 37 | C | -2.6297868 | -6.3602223 | 0.3637012 |
| 38 | C | -1.5807854 | -7.0729111 | 0.7854388 |
| 39 | H | -3.4829731 | -6.2180872 | 1.0279104 |
| 40 | H | -1.5567803 | -7.5209337 | 1.7756275 |
| 41 | H | -0.7085342 | -7.2394536 | 0.1545267 |

**Table S6.** Cartesian coordinates (in Å) of oxyluciferin form, *oxy-***5**(phenolate)

| No | atom | x | y | z |
| --- | --- | --- | --- | --- |
| 1 | C | 3.1090843 | 0.9111975 | -0.2294898 |
| 2 | S | 2.4566557 | 2.6099091 | -0.2142354 |
| 3 | N | 4.4196613 | 0.7991572 | -0.1323397 |
| 4 | C | 4.1550001 | 3.238104 | -0.0578305 |
| 5 | H | 4.4216882 | 3.8777573 | -0.904109 |
| 6 | H | 4.2841876 | 3.8086098 | 0.8664421 |
| 7 | C | 2.240704 | -0.2025543 | -0.3391628 |
| 8 | C | 0.8652635 | -0.1193765 | -0.4406805 |
| 9 | H | 2.7444079 | -1.1648307 | -0.3370439 |
| 10 | H | 0.4267653 | 0.8799404 | -0.4370767 |
| 11 | C | -0.0775416 | -1.1792416 | -0.5516966 |
| 12 | C | -1.454924 | -0.8751316 | -0.6435217 |
| 13 | H | -1.7590443 | 0.1719309 | -0.6259884 |
| 14 | C | -2.4436714 | -1.848651 | -0.7555419 |
| 15 | C | -3.8329235 | -1.4992134 | -0.8425927 |
| 16 | C | -2.083096 | -3.2575234 | -0.7891885 |
| 17 | C | -4.7965429 | -2.4552783 | -0.9524394 |
| 18 | H | -4.1001668 | -0.4424279 | -0.8174298 |
| 19 | C | -3.0694201 | -4.2408415 | -0.9208437 |
| 20 | C | -4.4782133 | -3.8828086 | -0.9873186 |
| 21 | H | -5.85092 | -2.1958336 | -1.0166211 |
| 22 | C | -0.6726004 | -3.551099 | -0.6813355 |
| 23 | H | -0.3549237 | -4.5885487 | -0.6722106 |
| 24 | C | 0.276501 | -2.5720912 | -0.570923 |
| 25 | H | 1.3222172 | -2.8567582 | -0.4895354 |
| 26 | C | 5.0854497 | 1.9962484 | -0.0340891 |
| 27 | O | -5.394832 | -4.7446462 | -1.072764 |
| 28 | C | -2.7557464 | -5.7239839 | -0.9927136 |
| 29 | H | -1.8197428 | -5.9153197 | -1.5328841 |
| 30 | H | -3.572082 | -6.1891114 | -1.5616958 |
| 31 | C | -2.7044837 | -6.3827413 | 0.3648626 |
| 32 | C | -1.6836188 | -7.1018554 | 0.8476061 |
| 33 | H | -3.5949417 | -6.2364639 | 0.9774247 |
| 34 | H | -1.7148788 | -7.5477346 | 1.8397832 |
| 35 | H | -0.7748532 | -7.2664192 | 0.2687614 |
| 36 | O | 6.2997549 | 2.1383569 | 0.0656642 |

**Table S7.** Cartesian coordinates (in Å) of oxyluciferin form, *oxy-***5**(ONa)

| No | atom | x | y | z |
| --- | --- | --- | --- | --- |
| 1 | C | 3.1192326 | 0.9497325 | -0.2268127 |
| 2 | S | 2.4933947 | 2.6417528 | -0.2158846 |
| 3 | N | 4.4101953 | 0.792429 | -0.1227479 |
| 4 | C | 4.2009113 | 3.2444491 | -0.0466872 |
| 5 | H | 4.4794768 | 3.8805259 | -0.8912199 |
| 6 | H | 4.3280504 | 3.8125524 | 0.8787975 |
| 7 | C | 2.2255573 | -0.1724489 | -0.3437964 |
| 8 | C | 0.8715713 | -0.0724996 | -0.4511676 |
| 9 | H | 2.7269373 | -1.1356675 | -0.3372963 |
| 10 | H | 0.4329067 | 0.9260161 | -0.4512367 |
| 11 | C | -0.0871 | -1.1491339 | -0.5665298 |
| 12 | C | -1.4461481 | -0.8406865 | -0.6611147 |
| 13 | H | -1.7556287 | 0.2037658 | -0.6473559 |
| 14 | C | -2.442043 | -1.8299671 | -0.7718956 |
| 15 | C | -3.8228975 | -1.4885078 | -0.8566718 |
| 16 | C | -2.0717245 | -3.2230452 | -0.7989149 |
| 17 | C | -4.7812869 | -2.4620457 | -0.9537102 |
| 18 | H | -4.105266 | -0.4372041 | -0.8390814 |
| 19 | C | -3.0702366 | -4.223419 | -0.9220836 |
| 20 | C | -4.4400464 | -3.8593908 | -0.9768313 |
| 21 | H | -5.8366442 | -2.2064637 | -1.0134976 |
| 22 | C | -0.6719926 | -3.5181189 | -0.6916018 |
| 23 | H | -0.3469044 | -4.5529037 | -0.680634 |
| 24 | C | 0.276242 | -2.5324261 | -0.5813618 |
| 25 | H | 1.3218179 | -2.8144234 | -0.4984068 |
| 26 | C | 5.1097912 | 1.9912112 | -0.0170257 |
| 27 | C | -2.7308622 | -5.7009187 | -0.9850657 |
| 28 | H | -1.7780131 | -5.8748096 | -1.498129 |
| 29 | H | -3.5056835 | -6.1857102 | -1.5959958 |
| 30 | C | -2.7034036 | -6.3657441 | 0.3730943 |
| 31 | C | -1.733057 | -7.165817 | 0.8291367 |
| 32 | H | -3.5543892 | -6.1406222 | 1.0186798 |
| 33 | H | -1.7730519 | -7.6056694 | 1.8229393 |
| 34 | H | -0.8554493 | -7.4011281 | 0.2282947 |
| 35 | O | 6.3183204 | 2.0889229 | 0.0903455 |
| 36 | O | -5.3919898 | -4.7513701 | -1.0488691 |
| 37 | Na | -6.7736916 | -6.196656 | -1.0964605 |

2.3. TDDFT calculations for excitations to the first three singlet-excited states of the oxyluciferin forms, *oxy*-**3**, *oxy*-**5**(phenolate), and *oxy*-**5**(ONa) using B3LYP/6-31+G(d)

2.3.1. Excitation of *oxy*-**3**

Excitation energies and oscillator strengths:

Excited State 1: Singlet-A 2.8479 eV 435.35 nm f=0.5794

HOMO -> LUMO 0.69804

Excited State 2: Singlet-A 3.3599 eV 369.02 nm f=0.0135

HOMO-2 -> LUMO -0.15825

HOMO-1 -> LUMO 0.61788

HOMO -> LUMO+1 -0.28615

Excited State 3: Singlet-A 3.4329 eV 361.17 nm f=0.0002

HOMO-4 -> LUMO 0.68563

HOMO-4 -> LUMO+2 0.13023

2.3.2. Excitation of *oxy*-**5**(phenolate)

Excitation energies and oscillator strengths:

Excited State 1: Singlet-A 2.4042 eV 515.69 nm f=1.2267

HOMO -> LUMO 0.70803

HOMO <- LUMO -0.13521

Excited State 2: Singlet-A 2.7618 eV 448.92 nm f=0.0025

HOMO-1 -> LUMO 0.69611

Excited State 3: Singlet-A 3.2171 eV 385.39 nm f=0.0308

HOMO-2 -> LUMO -0.42334

HOMO -> LUMO+1 0.55447

2.3.3. Excitation of *oxy*-**5**(ONa)

Excitation energies and oscillator strengths:

Excited State 1: Singlet-A 1.8310 eV 677.14 nm f=0.0024

HOMO -> LUMO 0.70338

Excited State 2: Singlet-A 2.6696 eV 464.43 nm f=0.8748

HOMO -> LUMO+1 0.69878

Excited State 3: Singlet-A 3.0472 eV 406.88 nm f=0.0004

HOMO-1 -> LUMO 0.70395

**S3. Reference**

(1) Frisch, M. J.; Trucks, G. W.; Schlegel, H. B.; Scuseria, G. E.; Robb, M. A.; Cheeseman, J. R.; Scalmani, G.; Barone, V.; Mennucci, B.; Petersson, G. A.; et al. Gaussian 09, Revision D.01. Gaussian, Inc.: Wallingford, CT 2004, p Gaussian 09, Revision D.01.

(2) Becke, A. D. Density-Functional Thermochemistry. III. The Role of Exact Exchange. *J. Chem. Phys.* **1993**, *98* (7), 5648–5652.

(3) Lee, C.; Yang, W.; Parr, R. G. Development of the Colle-Salvetti Correlation-Energy Formula into a Functional of the Electron Density. *Phys. Rev. B* **1988**, *37* (2), 785–789.

(4) Stephens, P. J.; Devlin, F. J.; Chabalowski, C. F.; Frisch, M. J. Ab Initio Calculation of Vibrational Absorption and Circular Dichroism Spectra Using Density Functional Force Fields. *J. Phys. Chem.* **1994**, *98* (45), 11623–11627.

(5) Dennington, R.; Keith, T.; Millam, J. GaussView, Version 5. *Semichem Inc. , Shawnee Mission, KS*. 2009, p Semichem Inc.
